# HPV16-LINC00393 Integration Alters Local 3D Genome Architecture in Cervical Cancer Cells

**DOI:** 10.3389/fcimb.2021.785169

**Published:** 2021-12-07

**Authors:** Xinxin Xu, Zhiqiang Han, Yetian Ruan, Min Liu, Guangxu Cao, Chao Li, Fang Li

**Affiliations:** ^1^ Department of Obstetrics and Gynecology, East Hospital, Tongji University School of Medicine, Shanghai, China; ^2^ Department of Obstetrics and Gynecology, Tongji Hospital, Tongji Medical College, Huazhong University of Science and Technology, Wuhan, China; ^3^ Shanghai Key Laboratory of Maternal Fetal Medicine, Shanghai First Maternity and Infant Hospital, School of Medicine, Tongji University, Shanghai, China

**Keywords:** cervical cancer, HPV integration, Hi-C, TAD boundary, gene expression

## Abstract

High-risk human papillomavirus (hrHPV) infection and integration were considered as essential onset factors for the development of cervical cancer. However, the mechanism on how hrHPV integration influences the host genome structure remains not fully understood. In this study, we performed *in situ* high-throughput chromosome conformation capture (Hi-C) sequencing, chromatin immunoprecipitation and sequencing (ChIP-seq), and RNA-sequencing (RNA-seq) in two cervical cells, 1) NHEK normal human epidermal keratinocyte; and 2) HPV16-integrated SiHa tumorigenic cervical cancer cells. Our results reveal that the HPV-LINC00393 integrated chromosome 13 exhibited significant genomic variation and differential gene expression, which was verified by calibrated CTCF and H3K27ac ChIP-Seq chromatin restructuring. Importantly, HPV16 integration led to differential responses in topologically associated domain (TAD) boundaries, with a decrease in the tumor suppressor KLF12 expression downstream of LINC00393. Overall, this study provides significant insight into the understanding of HPV16 integration induced 3D structural changes and their contributions on tumorigenesis, which supplements the theory basis for the cervical carcinogenic mechanism of HPV16 integration.

## Introduction

Cervical cancer is the second most common type of cancer in women worldwide with nearly 604,000 new cases diagnosed and 342,000 deaths in 2020 (Sung et al., 2021). High-risk human papillomaviruses (hrHPVs) such as HPV16, 18, and 31 were recognized as the essential factors to trigger tumorigenesis (Xia et al., 2017). Of these hrHPVs, HPV16 is responsible for approximately 50% of cervical cancer cases (Franceschi, 2021). Mechanically, the integration of HPV is considered to be a crucial event in promoting cervical carcinogenesis *via* alternating the transcription (Jeon and Lambert, 1995; Pett and Coleman, 2007) and chromosome instability of the host (Akagi et al., 2014). On one hand, the integration-targeted cellular genes in combination with the invariably retained and expressed oncoproteins E6/E7 can disrupt cervical epithelial cells cycle to immortalize cells and thus inducing the development of cervical cancer (Malanchi et al., 2002; Zhang et al., 2016; McBride and Warburton, 2017; Yeo-Teh et al., 2018). On the other hand, the integration of HPV may also cause chromosome instability and induce gene rearrangement and copy number variation (Duensing and Münger, 2004; PETT et al., 2004).

Although molecular mechanisms underlying acquired cervical cancer are extensively studied, how the 3D chromatin landscape responds to the integration of hrHPV is still not fully understood. For this reason, high-throughput chromosome conformation capture (Hi-C) technology has been developed to describe chromosome 3D structure. Hi-C could separate chromosomes into two different compartments *via* principal component analysis (Lieberman-Aiden et al., 2009). Compartment A represented transcriptional activation, while compartment B represented transcriptional inhibition. With the advance of Hi-C resolution, newly regions called topologically associating domains (TADs) (Dixon et al., 2012) were identified, which occur preferentially within defined and stable regions of the genome and are conserved among various tissues (Pope et al., 2014). TADs are separated by insulating proteins like the CCCTC-binding factor (CTCF) and which build a framework for contacts of regulatory TAD boundary and gene expression (Seitan et al., 2013; Zuin et al., 2014). The general activity within a given TAD can be influenced by its epigenomic state. Moreover, genes are most often regulated by enhancers located within the same TAD. To study these chromatin interactions, the combination of multiple capture assays such as Hi-C, assay of transposase accessible chromatin sequencing (ATAC-seq) (Wang et al., 2020), whole-genome sequencing (WGS) (Adeel et al., 2021), RNA sequencing (RNA-seq) and/or chromatin immunoprecipitation sequencing (ChIP-seq) (Zhang et al., 2020) has been developed.

Up to date, several studies have characterized the structural variations of HPV-driven cervical cancer within certain integration hot spots (Cao et al., 2020), or by examining the genome-wide interactions using more unbiased approaches (Adeel et al., 2021; Groves et al., 2021). Investigating chromatin structure in cancer has the potential to identify candidate biomarkers, since the organization of the chromatin is often disturbed in cancer (Barutcu et al., 2015). Despite the contributions of previous studies, differences in genome-wide chromatin structure between normal epithelial cells and tumorigenic cervical cancer cells still need to be explored. In the present study, in order to characterize different scales of genome organization during cervical cancer development, we have used NHEK normal human epidermal keratinocyte and SiHa tumorigenic cervical cancer cells and performed genome-wide Hi-C, ChIP-seq as well as RNA-seq to identify structural variations, specifically TAD boundaries. We interpreted a correlation among chromatin structure, epigenetic landscape, gene expression, and HPV-LINC00393 integrated loci in SiHa cells. The results will help us to get a better insight into HPV-LINC00393 integration in cervical carcinogenesis.

## Methods

### Cell Culture

Human cervical cell lines SiHa (ATCC HTB-35) and Ect1/E6E7 (ATCC CRL-2614) were obtained from the American Type Culture Collection (ATCC). Normal human epidermal keratinocytes (NHEK) were purchased from PromoCell (C-123003; Germany). All the cells were authenticated by short tandem repeat (STR) tests. SiHa/NHEK and Ect1/E6E7 were grown in MEM and EMEM (MEM plus NEAA) supplemented with 10% fetal bovine serum (Gibco, NY, USA), and 1% penicillin/streptomycin (Gibco, NY, USA). Cells were grown in a humidified 5% CO_2_ incubator at 37°C.

### RNA Extraction, RNA-seq, and Data Analysis

Total RNA was extracted using mirVana™ miRNA Isolation Kit (AM1561, Ambion^®^). RNA sequencing (RNA-seq) was performed by Annoroad Gene Technology Co., Ltd. (Beijing, China) (Li et al., 2019). For each sample, 3 μg of total RNA were used as initial material to generate sequencing libraries using the NEBNext^®^ Ultra^™^ Directional RNA Library Prep Kit according to the manufacturer’s recommendations. After the library was constructed, a series of processes were performed to ensure the quality of the library. Then, paired-end sequencing was performed on a single lane of Illumina HiSeq X Ten platform (Illumina, San Diego, CA, USA), with PE150 setting, producing 250 bp reads per end, according to manufacturer’s instructions.

After removing contaminated reads for adapters and low-quality reads, Bowtie2 (v2.2.6) was used for building the genome index, and clean data and was then aligned to the reference genome using HISAT2 v2.1.0. The Integrative Genomics Viewer (IGV) was used to view the mapping result (heatmap, histogram, and scatter plot). Reads count for each gene in each sample were counted by HTSeq v0.6.0, and FPKM (Fragments Per Kilobase Millon Mapped Reads) was then calculated to estimate the expression level of genes in each sample. DESeq2 v1.6.3 was designed for differential gene expression analysis between two samples with biological replicates under the theoretical basis that obeys the hypothesis of the negative binomial distribution for the value of count. DESeq2 was used to estimate the expression level of each gene in per sample by the linear regression, and then calculate the *p*-value with Wald test. Genes with *q*  ≤ 0.05 and |log_2__ratio| ≥ 1 were considered differentially expressed genes (DEGs). The Gene Ontology (GO) enrichment of DEGs was implemented by the hypergeometric test, in which the *p* value is calculated and adjusted as *q* value, and the data background is genes in the whole genome. GO terms with *q*  < 0.05 were considered to be significantly enriched. The Kyoto Encyclopedia of Genes and Genomes (KEGG) enrichment of DEGs was implemented by the hypergeometric test, in which the *p* value was adjusted by multiple comparisons as *q* value. KEGG terms with *q*  < 0.05 were considered to be significantly enriched.

### High-Throughput Chromosome Conformation Capture (Hi-C) Sequencing

Hi-C sequencing (Hi-C-seq) was performed by Annoroad Gene Technology Co., Ltd. (Beijing, China). Cells were first crosslinked and then were lysed under ice condition to the extracted DNA. After quantification, the final sequencing library was diluted to 1 ng/μl. StepOnePlus™ Real-Time PCR system was used for qPCR to accurately quantify the concentration of the library. The TruSeq PE Cluster Kit v3-cbot-HS (Illumia) reagent was used to generate clusters on the cBot. After that, the library was sequenced on Illumina HiSeq X Ten platform with PE150 setting.

For mapping and interaction identification, Bowtie2 (v2.2.6) was used to map the sequenced reads to the Arabidopsis TAIR10 genome. Multiple mapped reads, unmapped paired-end reads, singleton reads, and PCR duplications were filtered by Hi-C Pro pipeline. The uniquely valid paired-end reads were kept for downstream analysis. ICE methods (Yaffe and Tanay, 2011) were used to remove different biases after building the raw contact matrices. For compartment A/B identification, the matrix2 compartment module of the cworld software was used to detect the compartment under 40 kb resolution contact matrix. Interaction distances which were below 1 MB were filtered. The lowess-smoothed average method was used to calculate the expected scores of the intra- and interaction matrix. The observed/expected ratio was log2 transformed. The patterns of chromosomal interactions were calculated at each pair of bins by using Pearson correlation, and then using this correlated matrix to do the principal component analysis. The first principal component’s eigenvalue was plotted that positive values are referred to as compartment A, which means “open chromatin”, and negative values are referred to as compartment B, also means “closed chromatin”. The gene density was defined by calculating each bin’s gene number (Lieberman-Aiden et al., 2009).

### ChIP-seq and Data Analysis

ChIP sequencing (ChIP-seq) was performed by Annoroad Gene Technology Co., Ltd. (Beijing, China). Cells were crosslinked with 1% formaldehyde for 10 min at room temperature and quenched with 125 mM glycine. The fragmented chromatin fragments were pre-cleared and then immunoprecipitated with Protein A + G Magnetic beads coupled with anti-H3K27ac (ab4729, Abcam, USA) antibody. After reverse crosslinking, ChIP and input DNA fragments were end-repaired and A-tailed using the NEBNext End Repair/dA-Tailing Module (E7442, NEB) followed by adaptor ligation with the NEBNext Ultra Ligation Module (E7445, NEB). The DNA libraries were amplified for 15 cycles and sequenced using Illumina NovaSeq 6000 with single-end 1 × 75 as the sequencing mode.

Raw reads were filtered to obtain high-quality clean reads by removing sequencing adapters, short reads (length <35 bp) and low quality reads using Cutadapt (v1.9.1) (Martin, 2011) and Trimmomatic (v0.35) (Bolger et al., 2014). Then FastQC (Kyi et al., 2018) was used to ensure high reads quality. The clean reads were mapped to the human genome (hg19) using the Bowtie2 (v2.2.6) (Langmead and Salzberg, 2012) software. Peak detection was performed using the MACS (v2.1.1) (Zhang et al., 2008) peak finding algorithm with 0.01 set as the *p* value cutoff. Annotation of peak sites to gene features was performed using the ChIPseeker R package (Yu et al., 2015).

### Bioinformatic Analysis

The protein–protein interaction (PPI) network was constructed using Search Tool for the Retrieval of Interacting Genes (STRING) (https://www.string-db.org/) and visualized with the Cytoscape software (Shannon et al., 2003). A combined score of >0.9 was retained in the further analysis. The Gene Expression Profiling Interactive Analysis (GEPIA) was used to assess the expression of KLF12 in the three major gynecological tumors (cervical cancer, endometrial cancer, and ovarian cancer) of the TCGA data (Tang et al., 2017). The threshold Fold-change and *p* value were set at 1.5 and 0.05 respectively to get the expression boxplot. The Human Protein Atlas database (HPA) (https://www.proteinatlas.org) was used to analyze protein expression of KLF12 between normal and cervical cancer tissues (Uhlén et al., 2015; Tebani et al., 2020).

### Public Genomic Data Analysis

CTCF ChIP-seq datasets for NHEK and SiHa cells were downloaded from the NCBI Gene Expression Omnibus (GEO; accession number: GSM733740 for NHEK, and GSE143026 for SiHa) (ENCODE Project Consortium, 2012; Edgar et al., 2002).

## Results

### HPV16 Integration on LINC000393 of Chromosome 13 in Cervical Cancer

**Figure 1:**
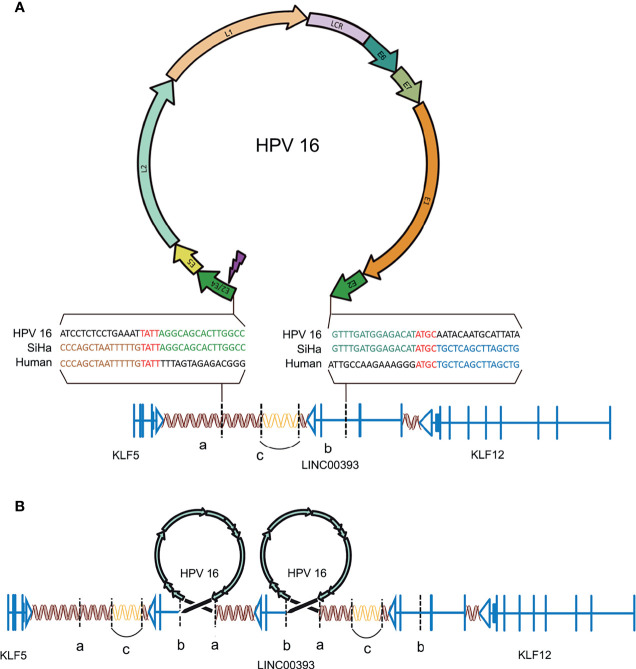
HPV16 integration in LINC00393 in SiHa cells. **(A)** An overview of HPV and the integration region. Purple lightning indicates the breakage of E2/E4. Double helix represents non-coding region, arrow represents the coding region, vertical line represents the exon, horizontal line represents intron and vertical dotted line represents the integration site. Gene model of HPV-LINC00393 integration (red ‘TATT’ and ‘ATGC’ indicate 5’ and 3’ micro-homologous fragments). **(B)** Structural variation of the integration region. Connected by HPV, the human genome between ‘a’ and ‘b’ contains a third exon, the second intron of LINC00393 and the desert gene are rearranged due to integration. The human genome, located between the two HPV genomes, lacks the C segment and is connected by itself (the sequence is represented with a blank double helix). ‘a’ means the integration site of 5’; ‘b’ means the integration site of 3’; ‘c’ means the lost gene segment during HPV16 integration.

The genes loci on the chromosomes of both SiHa and HPV16 were analyzed according to the UCSC database (http://genome.ucsc.edu/). There is a fragment of HPV16 (coordinates from 3384 to 7906/1–3132, length 7,654 bp) integrated on chromosome 13 at genomic coordinates 7378870–74087558 in the human genome (**Figure 1A**). Parts of the HPV16 E2 gene, along with the complete E4, E5, L2, L1, E6, E7, and E1 genes, were integrated into the genome of the SiHa cells. A microhomologous “AGTC” fragment was present upstream and a microhomologous “TATT” fragment was present downstream of the HPV integration. The 3’ integration locus of HPV16 is located on the second intron of LINC00393 gene, which is a high frequency integration site for HPV16 (Qiu et al., 2021), while the 5’ integration site is located on the intragenic region.

As a result, HPV16 integration leads to a recurrent pattern of DNA amplifications, with two insertional breakpoints directly flanked a twice-amplified segment (**Figure 1B**) (Akagi et al., 2014). This structural alteration is mostly represented by the rearrangements adjacent to the integration sites that leave the two HPV16 integration fragments sharing the same transcriptional orientation of LINC00393. Therefore, the analysis validated the integration status and pattern of HPV16 in SiHa cells and showed the alterations in the chromosomal structure.

### HPV-LINC00393 Integration Altered Local 3D Genome Structure in Cervical Cancer

**Figure 2:**
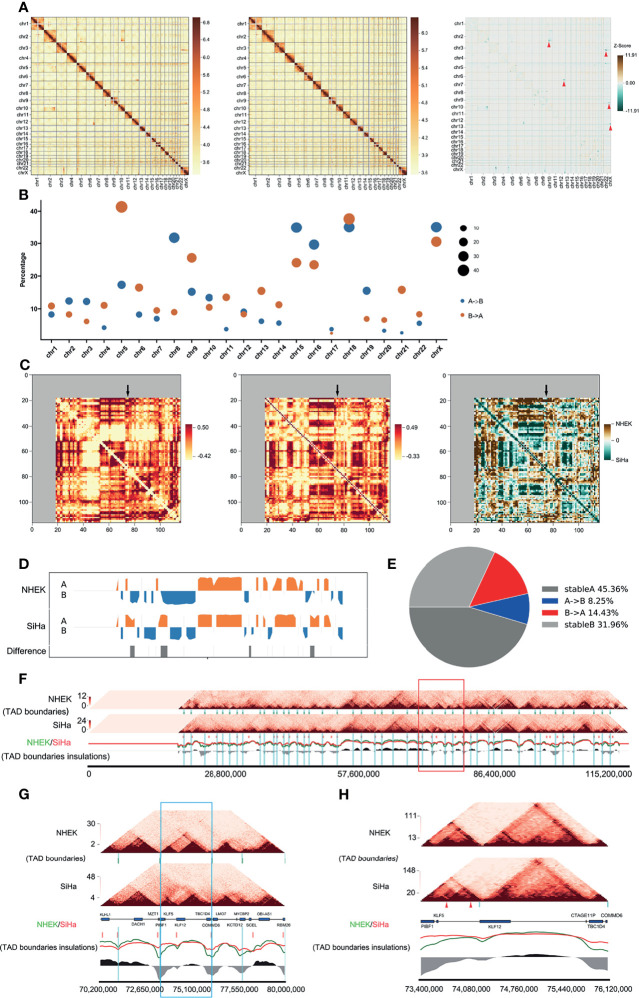
HPV16-LINC00393 integration altered local 3D genome structure of SiHa cells. **(A)** Heatmaps of the genome-wide chromosomal profiles of two cell lines (left: SiHa, right: NHEK) and their subtraction image (SiHa–NHEK). The red arrows show the structure alternations in SiHa. **(B)** Bubble diagrams showing percentage of switched compartments. Sizes of bubbles indicate the number of changed compartment. **(C)** Heatmaps indicate contact correlation matrix for chromosome 13. Left: NHEK; middle: SiHa; right: a reduced interaction matrix between SiHa and NHEK. **(D)** The compartment of the chromosome 13. NHEK lies at the top and SiHa lies at the bottom. Compartment A is in orange and compartment B is in blue. **(E)** The pie chart indicates the compartment change in chromosome 13 of two cell lines. The proportion of compartment B in NHEK which is turned into compartment A in SiHa is shown in red and the opposite situation is in blue. **(F)** The local comparison of TADs in SiHa and NHEK, including the comparison of heat maps, TAD boundaries and insulations. The short green vertical line shows the TAD boundary of NHEK and the short red one shows the TAD boundary of SiHa. The long blue vertical line is the TAD boundary of NHEK, which is used as a reference. The red (SiHa) and green (NHEK) wavy lines indicate the insulation scores of the two cells. The black and grey areas at the bottom represent the difference between NHEK and SiHa insulation scores. The red box represents the five TAD boundaries near the integration area. **(G)** The five TADs near the integration area shown in the red box of 2F. The blue box is the TAD boundaries in the integrated region. This TAD has a new TAD boundary and is divided into two smaller TADs in SiHa. **(H)** The details of the blue box in 2G. The reference gene above the scale is from NCBI (Chr13, 73400000–746120000). A new TAD boundary appears in SiHa shown by a blue vertical band. The genome integration sites of SiHa are triangulated in red. The TAD on the left has a tendency to split into two sub-TAD structures.

To get a better understanding of the structural variations caused by HPV16 integration, we compared the Hi-C-seq data of the cervical cancer SiHa and normal human epidermal keratinocyte NHEK cells. After filtering out the same compartments (AA, BB) and the different (AB, BA) linkers, approximately 90.8 million for NHEK and 153.7 million for SiHa reads were obtained (**Table S1**). The numbers of valid reads for NHEK and SiHa were 35,391,673 and 47,448,435, respectively. The visualized overall heatmap revealed higher order genomic organization (**Figure 2A**). The SiHa heatmap was consistent with that of the NHEK cells, except for some obvious differential organizations, which were possibly caused by cervical cancer itself. Next, we checked the A/B compartments and found that 71.2% of the total genome remained conserved between NHEK and SiHa cells, with only 13.1 and 15.7% of the annotated genome changing from A to B or B to A compartments, respectively. We also observed the A/B compartment switching at a high proportion in chromosomes such as chromosomes 2, 3, 6, and 9 (**Figure 2B**).

To investigate the differential interactions near the HPV16-LINC00393 locus, we compared the 3D structures of chromosome 13 in SiHa and NHEK cells. It showed that some genomic regions exhibited differential variations, especially around the integration locus (**Figure 2C**). Further comparison at a resolution of 1 MB disclosed several changes in the compartments composition in SiHa cells. Moreover, SiHa cells exhibited a higher degree of chromatin openness, which may facilitate gene transcriptional regulation (**Figure 2D**). A/B compartments analysis showed that 77.3% of the annotated chromosome 13 remained unchanged between the NHEK and SiHa cells, with only 8.3 and 14.4% changing from A to B or B to A compartments, respectively (**Figure 2E**).

TADs are important stable regulatory units, whose alterations are closely related to tumor development (Valton and Dekker, 2016). To identify the different TAD boundaries between SiHa and NHEK, TAD boundaries were detected at a resolution of 40 kb in each cell lines. The number (1,196 and 1,443 TAD boundaries for SiHa and NHEK, respectively) and average size of TAD boundaries were slightly different within the two cells (**Figure S1**). Among the identified 2,332 TAD boundaries, 307 TAD boundaries were overlapped, 889 and 1,136 were unique to SiHa and NHEK, respectively (**Figure S2**). Next, we characterized the specific changes of TAD boundaries on chromosome 13. It showed that HPV16-LINC00393 integration generated a longer chromosome, together with alterations on TAD boundaries (**Figure 2F**). After comparing the TAD boundaries around the integration site, a newly TAD boundary was observed in SiHa, along with the loss of normal interaction (**Figure 2G**). Further analysis suggested that HPV16-LINC00393 integration has induced the split of the TAD, which thereby influenced the original interaction (**Figure 2H**).

### HPV-LINC00393 Integration Altered TAD Insulation and Enhancer–Promoter Interaction

**Figure 3:**
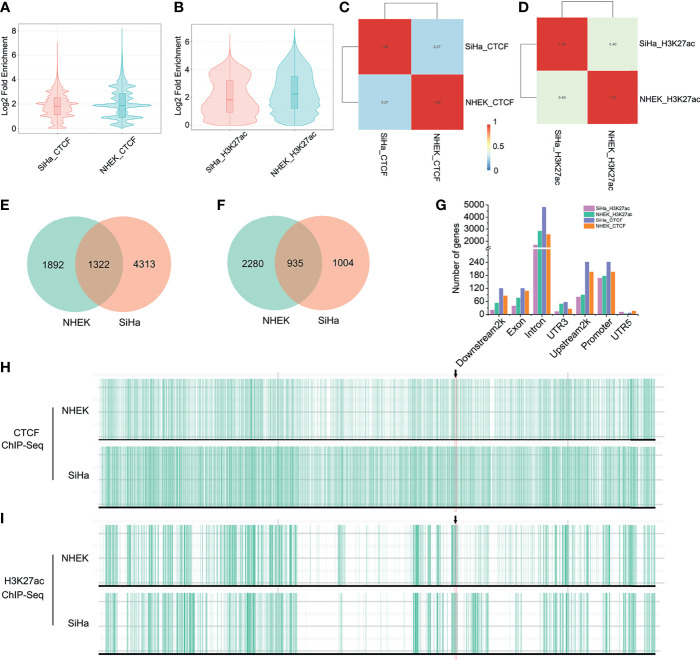
HPV integration altered TAD insulation and enhancer-promoter interaction. **(A)** Genome-wide distribution of CTCF in SiHa and NHEK. **(B)** Genome-wide distribution of H3K27ac in SiHa and NHEK. **(C)** The binding locations of CTCF in SiHa and NHEK. **(D)** The binding locations of H3K27ac in SiHa and NHEK. **(E)** The number of unique and shared CTCF between SiHa and NHEK. **(F)** The number of unique and shared H3K27ac between SiHa and NHEK. **(G)** The number of CTCF and H3K27ac targeted genes located in different gene regions. **(H, I)** CTCF and H3K27ac ChIP-seq tracks for the chromosome 13 of SiHa and NHEK.

Because the TAD fusion was associated with CCCTC-binding factor (CTCF) changes, we applied ChIP-seq to detect CTCF profiles in SiHa and NHEK cells. As is shown, genome-wide CTCF distribution did not differ significantly between the two cells (**Figure 3A**). However, the binding locations of CTCF in SiHa only matched approximately 27% of the TAD boundaries in NHEK cells (**Figure 3C**). As for the HPV-LINC00393 integrated chromosome 13, the number of CTCF was 3,214 and 5,635, respectively, with 1,322 CTCF being shared between the two cells (**Figure 3E**). The number of CTCF targeted genes located in the seven different gene regions was also discrepant (**Figure 3G** and **Table S2**). Furthermore, we confirmed that SiHa cells displayed intense CTCF binding at the TAD boundary of LINC00393 locus, with a weak binding found in the NHEK cells (**Figure 3H**).

Since histone epigenetic modifications can also disturb host 3D genome structure, we then performed H3K27ac ChIP-seq in the two cells. H3K27ac distribution was narrow in SiHa cells, indicating reduced enhancer activity compared to NHEK cells (**Figure 3B**). Meanwhile, the binding locations of H3K27ac in SiHa matched approximately 40% of that in NHEK (**Figure 3D**). Using a *q* value cutoff of 0.05, we found that approximately 48% of the H3K27ac regions in SiHa and approximately 29% of the H3K27ac peaks in NHEK were overlapped (**Figure 3F**). The number of H3K27ac modified genes was decreased in the seven different gene regions of SiHa cells (**Figure 3G** and **Table S2**). Furthermore, it showed that SiHa cells exhibited weak H3K27ac binding at the TAD boundary of the LINC00393 integration site, whereas an intense binding was observed in the NHEK cells (**Figure 3I**).

### Effect of HPV16-LINC00393 Integration on Gene Expression

**Figure 4:**
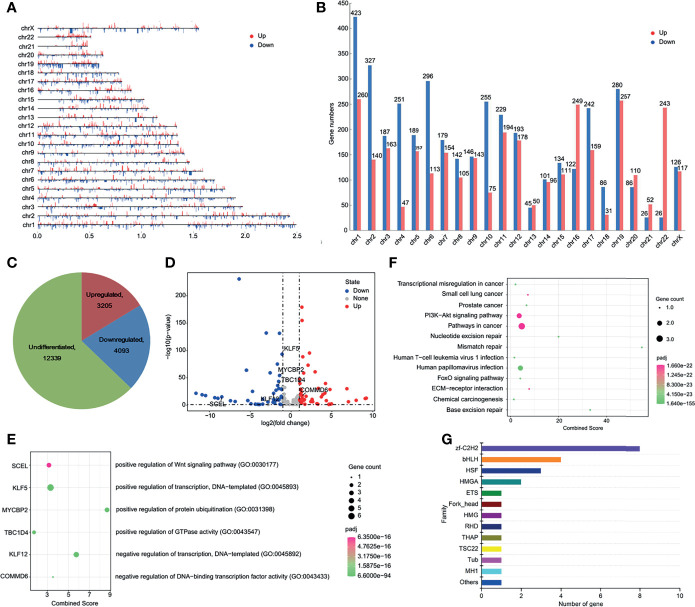
HPV16-LINC00393 integration altered gene expression in SiHa cells. **(A)** The distribution of differential expressed genes in each chromosome. **(B)** The statistical graph showed the numbers of differential expressed gene in each chromosome. **(C)** The distribution of transcription changes in SiHa as compared with Ect1/E6E7 by RNA-seq. **(D)** Volcano map showing the differentially expressed genes within the chromosome 13 between SiHa and Ect1/E6E7. **(E)** Integrated analysis of HPV16-LINC00393 integration associated genes (left panel) and functional annotation (right panel). **(F)** KEGG enrichment analysis based on the Metascape platform. **(G)** Analysis of differential transcription factor. The vertical axis shows the different transcription factor families and the horizontal one shows the number of genes annotated to the transcription factor family.

Chromatin translocation was reported to alter host gene expression and lead to cancer development (Gryder et al., 2021). To investigate the effect of translocation induced changes of TAD boundary on the expression of surrounding genes, we performed the transcriptome analysis of SiHa and normal cervical cell Ect1/E6E7. The overall distribution of the differentially expressed genes (DEGs) was shown in **Figures 4A–C**. Next, we investigated the effect of LINC00393 integration on the expression of genes present on chromosome 13. A total of 74 genes were characterized as DEGs, with 37 downregulated and 37 upregulated (**Figure 4D** andd **Table S3**). The nearby regions of LINC00393 such as KLF5, KLF12, TBC1D4, MYCBP2, and SCEL showed downregulated expression, while COMMD6 appeared as an upregulated gene (**Figure 4E**). Further analysis revealed that these DEGs were potentially involved in tumorigenesis-related, DNA repair, and HPV infection pathways (**Figure 4F**). Because transcription factor (TF) is an important determinant responsible for transcriptional activation of genes involved in tumorigenesis, we conducted TF analysis based on the DEGs. As is shown, the most enriched TF belonged to C2H2 type zinc finger (zf-C2H2) family (**Figure 4G**).

### HPV16-LINC00393 Integration Downregulated Tumor Suppressor Gene KLF12

**Figure 5:**
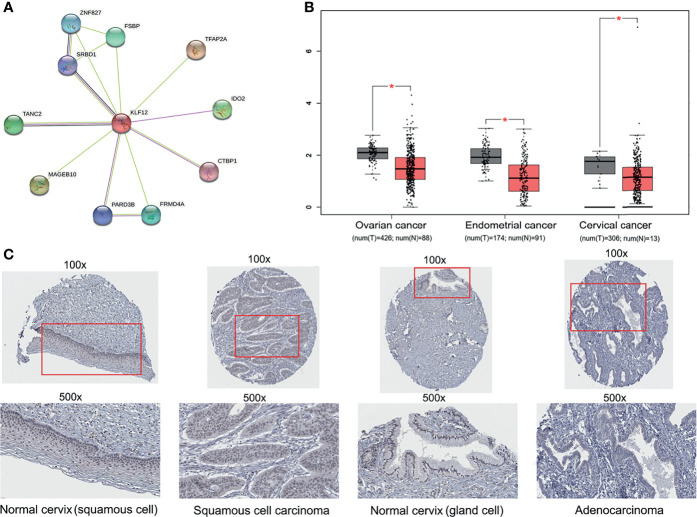
Multi-database analysis for the function and expression of KLF12. **(A)** The protein–protein interaction (PPI) network of KLF12 was mapped based on the STRING database. The purple edges were described as experimentally determined known interactions, the light green edges meaning textmining and the black ones meaning ‘co-expression’. The filled nodes mean their 3D structures are known or have been predicted, and the empty ones mean unknown. **(B)** The expression of KLF12 in gynecological tumors were shown based on the GEPIA and TCGA database. **(C)** Immunohistochemical results of KLF12 in normal cervix and cervical cancer from HPA database. *p < 0.05.

The tumor suppressor gene KLF12 is a member of zf-C2H2. However, whether KLF12 plays a direct role in cervical cancer and whether its inhibition can promote cervical tumorigenesis remains unclear. To address this, we used the STRING database and mapped the protein–protein interaction network of KLF12 (**Figure 5A**). Among the top ten proteins that interact with KLF12, CTBP1 was reported to be a key mediator for the transcriptional inhibitory role of KLF12 (McConnell and Yang, 2010). We next used the GEPIA website based on the TCGA database and analyzed the expression of KLF12 in the three major gynecological tumors. Compared with the healthy women, KLF12 expression was significantly downregulated in patients with ovarian cancer, endometrial cancer, and cervical cancer (**Figure 5B**). Furthermore, we used the HPA database to validate KLF12 expression in cervical cancer and normal cervical tissues. Decreased expression of KLF12 was observed in the nucleus of both cervical squamous cell carcinoma tissue and adenocarcinoma tissue (**Figure 5C**). These data indicate that KLF12 may connect with cervical tumorigenesis and have the potential to predict the onset of cervical cancer in HPV16-LINC00393 integrated patients.

## Discussion

Cervical cancer has become one of the most popular cancers in women (Sung et al., 2021) with HPV infection and integration as the main cause (Gryder et al., 2021). Previous studies have mostly analyzed the impact of HPV integration on the one-dimensional structure (Kadaja et al., 2009; Matovina et al., 2009) rather than three-dimensional structure. It remains unclear how such integration has influenced the host genome structure and transcription regulation. For this purpose, our study combines Hi-C-seq, ChIP-seq and RNA-seq to investigate the changes in 3D structure of HPV16 integrated SiHa cells. We found that chromosome 13 exhibited significant genomic variation and differential expression densities, with a correlation found between 3D structural change and gene expression. Enrichment analysis suggested that the dysregulated genes were mainly involved in controlling cervical cancer-related pathways. Therefore, this study advances our knowledge of the HPV16 integration on chromosome architecture changes and cervical tumorigenesis.

Previous studies indicated that SiHa has two copies of HPV16 DNA (Diao et al., 2015) thereby theoretically possessing four virus–human junctions, whereas the DNA sequencing results showed there were only two virus–human junctions (Baker et al., 1987; El Awady et al., 1987), suggesting the two integrated HPV might have the same junction and partially overlapped with each other at the integration site (Akagi et al., 2014). Importantly, one of the integration sites was found located at the second exon of LINC00393 on chromosome 13, which is likely to impact the expression of the surrounding genes (Qiu et al., 2021). Due to LINC00393 being a high-frequency site for HPV integration (Hu et al., 2015; Qiu et al., 2021) and no HPV episomes having been found in SiHa (Friedl et al., 1970), this makes SiHa a reliable research material for exploring the underlying pathogenicity mechanism of HPV16 integration in cervical cancer development.

Compared with the normal epidermal keratinocyte NHEK, the HPV16-integrated SiHa cells exhibited more 3D structure variations following Hi-C-seq analysis. The findings were consistent with those from Dixon et al., in which the variations might have been caused through chromatin translocation (Dixon et al., 2018). The newly generated TAD boundaries tend to divide the original ones into different subTAD boundaries, with disordered internal structures near the integration area. The result is also consistent with previous studies that HPV integration that led to significant 3D structural changes on the chromosome of the integration locus (Cao et al., 2020). Although a lot of DEGs were down-regulated in SiHa cells, much greater number of compartment A was detected, which can be explained as more genes involved in compartment B or structural changes in compartment A.

Our study found a newly generated TAD boundary in the LINC00393-integrated site of SiHa cells after integrated analysis of Hi-C and ChIP data. TADs are highly conserved domains across the genome separated by insulators such as CTCF which restricts the action of regulatory elements and genes. Meanwhile, the changes of H3K27ac can modulate between enhancer and promoter for the interactions and disrupt host TAD structure to rewire the regulatory landscape of genes (Melo et al., 2020). As a result, the genes that are near the overlap of CTCF and H3K27ac peaks have dysregulated expression. As expected in our study, the SiHa cells displayed an intense CTCF while having weak H3K27ac bindings at the TAD boundary of the LINC000393 integration site and changed the expression of the neighboring gene such as KLF12. The results imply that the generated TAD boundary might have introduced new CTCF modifications and thus obstructing the enhancer–promoter interaction.

It is interesting to note that the transcriptional regulatory (TF) protein of C2H2 zinc finger (zf-C2H2) was the most enriched TF among the differentially expressed TFs in SiHa cells (Liu et al., 2020). Zf-C2H2 proteins are human virus transcriptional regulators and can bind to DNA, RNA, and proteins. KLF12, the downstream gene of LINC00393 integration locus, is a member of the zf-C2H2 family. KLF12 normally serves as a transcriptional repressor through its interaction with the C-terminal binding protein (CtBP) (McConnell and Yang, 2010). In our study, it is found that KLF12 was mainly distributed in the nucleus with a reduced expression in cervical tumor tissues according to HPA database. Besides, the expression of KLF12 was also decreased in ovarian cancer and endometrial cancer, suggesting its role as a biomarker for gynecological tumor monitoring.

KLF12 has been reported as an important TF that participates in the tumorigenesis of various cancers. In some cases, KLF12 was overexpressed and served as a tumor suppressor, for example, in bladder cancer (Tang et al., 2021) and lung cancer (Godin-Heymann et al., 2016). In other cases, KLF12 expression was correlated positively with disease severity such as in colorectal cancer (Bai et al., 2021), lung cancer (Mao et al., 2020), ovarian cancer (Mak et al., 2017), and endometrial cancer (Ding et al., 2019), indicating its tumor-promoting effect. In the present study, both the expressions of KLF12 in SiHa cells and tumor tissues based on the HPA database were significantly down-regulated compared with those in normal control cells or clinical samples. In addition, the decreased expression of KLF12 is attributed to the 3D structural changes in HPV-integrated chromosome 13, which can partially explain the carcinogenic mechanism for HPV integration. Nevertheless, our results support the former point of view.

Our study has some limitations. Firstly, the interaction matrices were binned at a resolution of 40 kb to identify TAD boundaries, while the loop structure only available at a resolution of 5 kb, according to the technological development of Hi-C. Thus, still higher resolution is needed for deep analysis to find out better results. Secondly, although a new TAD boundary was formed due to HPV16-LINC00393 integration, it still needs to be verified by using clinical samples that have the same integration loci. Thirdly, this study has used two cells for the controls, the one is the NHEK normal human epidermal keratinocyte for Hi-C-seq, and the second is the Ect1/E6E7 cervical normal cell for RNA-seq. Despite of the same origin from ectoderm for the two cells, their distribution in the human body is different. Hence, there may be some deviations in the final accuracy of the 3D genome structure.

In summary, our study applied multi-omics sequencing analysis and demonstrated that HPV16-LINC00393 integration altered the 3D chromatin landscape and led to the enrichment of genome variations and gene expression changes in SiHa cell lines. The correlation between gene expression and TAD boundary change, enhancer–promoter interaction change, and also ectopic CTCF binding was also elucidated. These findings shed light on the important role of the 3D genome structure in cervical carcinogenesis when investigating the effects of HPV16 integration.

## Data Availability Statement

All raw sequence data (Hi-C-seq, RNA-seq and ChIP-seq) generated in this study have been submitted to the NCBI BioProject database (https://www.ncbi.nlm.nih.gov/bioproject/) under accession number PRJNA768938. All datasets generated for this study are included in the manuscript and/or the **Supplementary Material**.

## Author Contributions

CL and FL designed research. XX, ZH, YR, ML, GC, CL, and FL analyzed data. XX and ZH wrote the paper. XX, CL, and FL revised the paper. All authors contributed to the article and approved the submitted version.

## Funding

This work was supported by the National Natural Science Foundation of China (81771529), the Special Fund Project of “Fundamental Research Funds for the Central Universities” of Tongji University (22120190214), the Shanghai Science and Technology Development Foundation (21S31905100), the East Hospital initial foundation (DFRC201917), and Shanghai Municipal Health Commission (20194Y0156).

## Conflict of Interest

The authors declare that the research was conducted in the absence of any commercial or financial relationships that could be construed as a potential conflict of interest.

## Publisher’s Note

All claims expressed in this article are solely those of the authors and do not necessarily represent those of their affiliated organizations, or those of the publisher, the editors and the reviewers. Any product that may be evaluated in this article, or claim that may be made by its manufacturer, is not guaranteed or endorsed by the publisher.
