# Supplementary material for: HPV16-LINC00393 Integration Alters Local 3D Genome Architecture in Cervical Cancer Cells
Source: Front Cell Infect Microbiol. 2021 Dec 7;11:785169. doi: 10.3389/fcimb.2021.785169 (PMC8691139; doi:10.3389/fcimb.2021.785169)
Supplement: Supplementary file 2 [file DataSheet_2.docx]

Supplementary Material

# Supplementary Figures


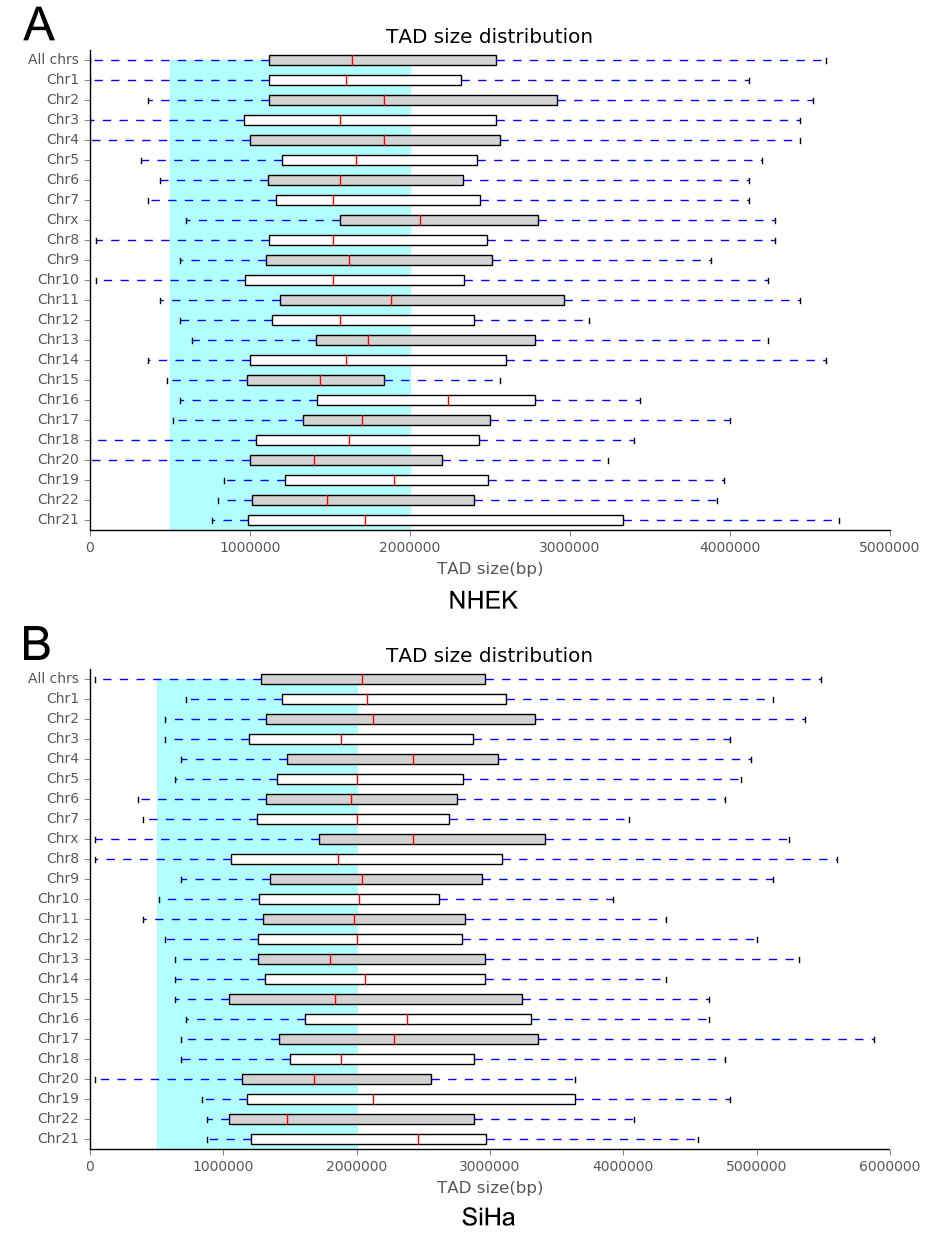


**Supplementary Figure 1.** TAD boundary size distribution of NHEK and SiHa cells. (A) NHEK. (B) SiHa.


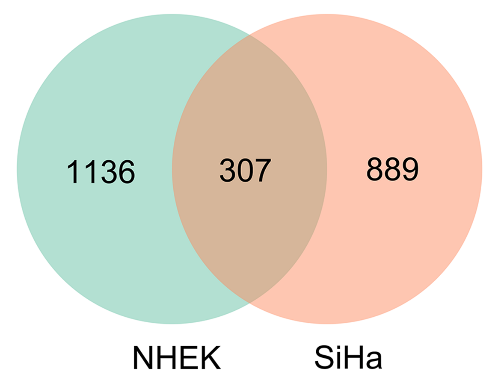


**Supplementary Figure 2.** Venn diagram showing the number of unique and shared TAD boundaries between SiHa and NHEK.
